# Endoscopic management of extensive ileocolic intussusception in Peutz-Jeghers syndrome is able to avoid surgery

**DOI:** 10.1055/a-2701-5162

**Published:** 2025-09-24

**Authors:** Abdeldjalil Sais, Elena De Cristofaro, Sophie Heissat, Jérôme Rivory, Jean-Christophe Saurin, Laura Calavas, Mathieu Pioche

**Affiliations:** 1 639305 Department of Gastroenterology, Groupement Hospitalier Portes de Provence, Montélimar, France; 2 9318 Gastroenterology Unit, University of Rome Tor Vergata, Rome, Italy; 3 Department of Pediatric Gastroenterology and Endoscopy, Hopital Femme mère enfants, Hospices Civils de Lyon, Lyon, France; 4 Department of Gastroenterology and Endoscopy, Hôpital Edouard Herriot, Hospices Civils de Lyon, Lyon, France

Peutz–Jeghers syndrome is a rare hereditary condition characterized by mucocutaneous pigmentation and Peutz–Jeghers hamartomatous polyps, predominantly affecting the small intestine 1 2 . It was first described by Peutz in 1921 and Jeghers in 1944 and 1949 3 . The risk of intussusception is estimated to be 44% by the age of 10 and about 50% by the age of 20 years old 4 , particularly for polyps larger than 15 mm.

Intussusception in Peutz–Jeghers syndrome requires a multidisciplinary approach. Nonoperative reduction may be attempted, though surgery is frequently necessary, particularly in the presence of complications such as bowel ischemia, perforation, or unsuccessful nonoperative reduction 5 .

Here, we illustrate a purely endoscopic management strategy, highlighting the essential procedural steps to effectively resolve extensive ileocolic intussusception caused by a large polyp, thereby entirely avoiding surgery.

A 14-year-old girl with genetically confirmed Peutz-Jeghers syndrome presented with recurrent abdominal pain, and imaging revealed an 8-cm ileo-colonic intussusception due to a 4-cm polyp ( Fig. 1 ). Endoscopic evaluation showed the large pedunculated polyp invaginated into the right colon through the ileocecal valve.

**Fig. 1:**
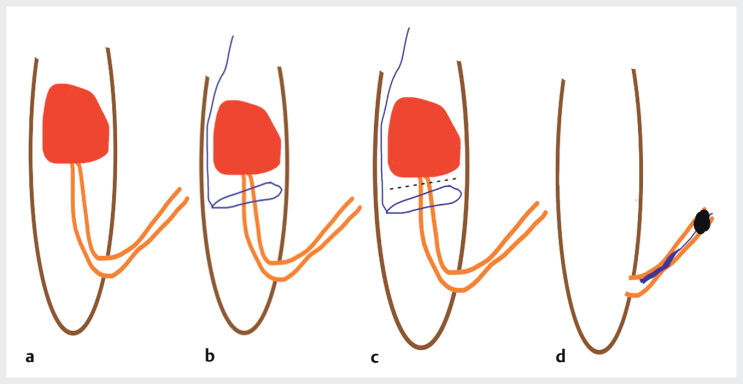
Schematic description of the procedure. **a** Initial aspect with an 8-cm ileo-colonic intussusception due to a 4-cm pedunculated polyp. **b** Placement of an endoloop at the polypʼs stalk base. **c** Piecemeal mucosectomy resection using a hot snare; immediate spontaneous desinvagination. **d** Multiple endoscopic clips were placed to reduce the risk of recurrent intussusception.

The endoscopic technique employed involved precise placement of an endoloop at the polyp's stalk base ( Fig. 1 **b, c** ) followed by piecemeal mucosectomy resection using a hot snare. Immediate spontaneous desinvagination of approximately 50 cm of the small bowel occurred following polyp removal ( Video 1 , Fig. 1 **d** ). Multiple endoscopic clips were then strategically placed to reduce the risk of recurrent intussusception by fixing the area of the previous resection with surrounding folds.

Endoscopic resolution of extensive ileocolic intussusception using endoloop and piecemeal resection, followed by clip placement to prevent recurrence in Peutz-Jeghers syndrome.Video 1

Histopathological examination confirmed that the resected polyp was a hamartoma without dysplasia.

This case demonstrates the technical feasibility and clinical advantages of an endoscopic-only approach to managing ileocolic intussusception in Peutz-Jeghers patients. This strategy offers a reliable alternative to surgical intervention, highlighting significant educational value for pediatric gastroenterologists and endoscopists.

Endoscopy_UCTN_Code_TTT_1AQ_2AD_3AB

## Notes

Endoscopy E-Videos https://eref.thieme.de/e-videos
*E-Videos* is an open access online section of the journal *Endoscopy* , reporting on interesting cases and new techniques in gastroenterological endoscopy. All papers include a high-quality video and are published with a Creative Commons CC-BY license. Endoscopy E-Videos qualify for HINARI discounts and waivers and eligibility is automatically checked during the submission process. We grant 100% waivers to articles whose corresponding authors are based in Group A countries and 50% waivers to those who are based in Group B countries as classified by Research4Life (see: https://www.research4life.org/access/eligibility/ ). This section has its own submission website at https://mc.manuscriptcentral.com/e-videos .
